# Patient-perceived barriers and facilitators to the implementation of a medication review in primary care: a qualitative thematic analysis

**DOI:** 10.1186/s12875-017-0707-0

**Published:** 2018-01-05

**Authors:** Mirella Carolin Uhl, Christiane Muth, Ferdinand Michael Gerlach, Goentje-Gesine Schoch, Beate Sigrid Müller

**Affiliations:** 1 Institute of General Practice, Goethe University Frankfurt, Theodor-Stern-Kai 7, 60590 Frankfurt am Main, Germany; 2 Scientific Institute for Benefit and Efficiency in Health Care (WINEG), Techniker Krankenkasse (TK), Bramfelder Straße 140, 22305 Hamburg, Germany

**Keywords:** Multimorbidity, Multiple chronic conditions, Polypharmacy, Patients, Primary health care

## Abstract

**Background:**

Although polypharmacy can cause adverse health outcomes, patients often know little about their medication. A regularly conducted medication review (MR) can help provide an overview of a patient’s medication, and benefit patients by enhancing their knowledge of their drugs. As little is known about patient attitudes towards MRs in primary care, the objective of this study was to gain insight into patient-perceived barriers and facilitators to the implementation of an MR.

**Methods:**

We conducted a qualitative study with a convenience sample of 31 patients (age ≥ 60 years, ≥3 chronic diseases, taking ≥5 drugs/d); in Hesse, Germany, in February 2016. We conducted two focus groups and, in order to ensure the participation of elderly patients with reduced mobility, 16 telephone interviews. Both relied on a semi-structured interview guide dealing with the following subjects: patients’ experience of polypharmacy, general design of MRs, potential barriers and facilitators to implementation etc. Interviews were audio-recorded, transcribed verbatim, and analysed by two researchers using thematic analysis.

**Results:**

Patients’ average age was 74 years (range 62–88 years). We identified barriers and facilitators for four main topics regarding the implementation of MRs in primary care: patient participation, GP-led MRs, pharmacist-led MRs, and the involvement of healthcare assistants in MRs. Barriers to patient participation concerned patient autonomy, while facilitators involved patient awareness of medication-related problems. Barriers to GP-led MRs concerned GP’s lack of resources while facilitators related to the trusting relationship between patient and GP. Pharmacist-led MRs might be hindered by a lack of patients’ confidence in pharmacists’ expertise, but facilitated by pharmacies’ digital records of the patients’ medications. Regarding the involvement of healthcare assistants in MRs, a potential barrier was patients’ uncertainty regarding the extent of their training. Patients could, however, imagine GPs delegating some aspects of MRs to them.

**Conclusions:**

Our study suggests that patients regard MRs as beneficial and expect indications for their medicines to be checked, and possible interactions to be identified. To foster the implementation of MRs in primary care, it is important to consider barriers and facilitators to the four identified topics.

**Electronic supplementary material:**

The online version of this article (10.1186/s12875-017-0707-0) contains supplementary material, which is available to authorized users.

## Background

The prevalence of multimorbidity rises with age and already affects more than 60% of patients above the age of 50 [1–3]. Multimorbidity is defined as the simultaneous occurrence of several acute or chronic illnesses and medical conditions in one person [4]. Increasing life expectancy can be expected to lead to a significant increase in its prevalence [5]. Multimorbidity is often associated with polypharmacy. No universally accepted definition of polypharmacy exists, but the term is generally used to describe patients taking five or more medicines per day [6, 7]. The number of patients taking five or more medicines increases with age, and the number prescribed 10 or more nearly doubles between the ages of 65 and 80 [8]. Additionally, about 24% of elderly patients are prescribed at least one potentially inappropriate medication [9]. As falls and hospital admissions often result from drug-disease interactions and adverse drug reactions, polypharmacy is a risk factor for adverse health outcomes [10–13]. Additionally, patients with polypharmacy often know too little about their drugs and have difficulties with treatment compliance [14].

A regular medication review (MR) defined as “a structured, critical examination of a patient’s medicines with the objective of reaching an agreement with the patient about treatment, optimising the impact of medicines, minimising the number of medication-related problems and reducing waste” can curb inappropriate polypharmacy [15]. MRs are also a first essential step towards gaining a comprehensive overview of a patient’s current medication [16]. Although evidence on the effect of MRs on hard clinical outcomes is lacking [17], a reduction in drug-related problems and the number of prescribed drugs has been demonstrated [18–20]. General practitioners (GPs) play a key role in MRs because of their generalist perspective and role in the coordination and continuity of care [21]. However, the workload of GPs hinders the use of MRs [22]. Pharmacists have also been involved in MRs, but their acceptance by patients varies considerably. High implementation rates for their recommendations (up to 86%) have been observed when the relationship between GPs and pharmacists has existed for some time [23, 24].

Although successful MRs require the active participation of patients, studies on patients’ attitudes to MRs are scarce [25]. Patients expect MRs to provide them with detailed information on their current medication, e.g. on necessity, and hope they will lead to simplified medication regimens. Not all patients feel the need to undergo an MR [26, 27], but those that have done see benefits in the enhancement of their knowledge about their medication, and the chance to ask about new drugs [15, 25, 28, 29]. Overall, little is known about patient-perceived barriers and facilitators to MRs in general practice.

Structured MRs are not regularly conducted in primary care in Germany. We therefore developed and tested a complex intervention involving GPs and their healthcare assistants (HCAs)^1^ to optimise and prioritise multiple medications in older patients with multimorbidity in primary care. The intervention included an MR and was generally feasible for health care professionals and patients during the pilot study [22]. After conducting the main study (publication in progress), to facilitate routine implementation of MRs in general practice, we have now conducted a qualitative barrier analysis to obtain patient perspectives on hindering and facilitating factors.

## Methods

### Design and setting

The study was conducted in primary care, where most prescriptions are managed. To include patients with reduced mobility and to support patient interaction, we investigated patients’ perspectives using two different approaches, namely 1) telephone interviews and 2) focus groups. Both relied on a semi-structured interview guide. The telephone interviews were conducted first.

### Participants

From January to February 2016, we recruited a convenience sample of patients from 17 general practices in the city and region of Frankfurt am Main. GPs from these practices belonged to “Forschungsnetzwerk Allgemeinmedizin Frankfurt” (ForN, Frankfurt research network of general practices) [30]. We asked GPs via e-mail to invite patients to participate in the study if they 1) were ≥60 years old, 2) had ≥3 chronic diseases, and 3) took ≥5 drugs per day. GPs were told about the study and its ultimate aim (to design an MR for primary care) and informed that travel expenses would be refunded for focus group participants. When a GP agreed to recruit patients, we sent him or her five consent forms and patient information sheets, with further copies available on request. Patients received written information on the study (general importance of medicines, polypharmacy as potential problem, and drug-related problems), the choice between telephone interview and focus group, and data protection regulations. After receiving written informed consent, their GP informed the study centre, the Frankfurt Institute of General Practice. The Institutional Review Board of the University Hospital Frankfurt/Main approved the study protocol in January, 2016 (No 443/15).

### Data collection

Patients were called up by the Frankfurt Institute of General Practice to arrange appointments for either a telephone interview or a focus group session to be carried out in February 2016. Patients had no previous association with the Institute. A preliminary interview guide was developed by BSM (academic GP, MD) and a student assistant (during the final term of his sociology studies), and revised after discussion with CM (principal investigator, MD, MPH) on the basis of her own research and existing literature [16, 22, 31]. The semi-structured interview guide covered the following subjects: patients with polypharmacy, design of a medication review (person in charge, benefits, difficulties) and services provided by health insurers (see Additional file 1). Conversations were structured but open, enabling patients to add their own comments [32]. At the beginning of both telephone interviews and focus groups, the two female interviewers (BSM, MCU) introduced themselves as staff from the Institute, but did not provide information on personal goals or academic background in order to minimise their influence on the conversation. BSM and MCU also provided background information on the study (see Additional file 1). BSM has considerable experience in conducting interviews and focus groups, and briefed MCU (doctoral candidate) carefully in advance. To get comprehensive insights into patients’ perspectives, we adapted the content of our interviews and focus groups by expanding on subjects that seemed important to them, and rewording and clarifying questions for better understanding. Subsequent interviews and focus groups were improved, based on field notes that were made when it had proven necessary to reword or clarify questions. Focus groups took place at the Institute. Patients did not receive payment but travel expenses were refunded. BSM and MCU conducted the focus groups and ensured that all important subjects were covered without interrupting the discussions. At the beginning of each focus group, BSM explained the procedure (talking and interacting with each other is encouraged, different attitudes are welcomed, others should be permitted to finish speaking). After each focus group, a short debriefing took place. Telephone interviews and focus groups were audio recorded.

### Data analysis

Telephone interviews and focus groups were anonymised and transcribed verbatim using f4transkript [33] software. Transcripts were not returned to interviewees for comment, correction, or feedback on the findings.

We used thematic analysis - a form of qualitative content analysis - to analyse the transcripts [34]. It contains the following phases: 1) familiarisation with the data; 2) generate initial codes; 3) search for themes; 4) review themes; 5) define and name themes; and 6) produce the report including a selection of illustrative data and quotations [34]. To explore the diversity of patient perspectives on barriers to and facilitators of the implementation of an MR, we analysed transcripts from telephone interviews and focus groups together. There was no evidence of any relevant differences in the findings between the two types of interviews, the same topics emerged from the respective transcripts.

BSM and MCU independently coded a sample of three transcripts and then checked for agreement on the codes. Afterwards, both coded the remaining transcripts. They then discussed the codes and emerging subjects, and reached a consensus by recoding or redefining them in case of disagreement. To minimise personal bias stemming from the differing disciplinary perspectives of the coders, the transition from codes to preliminary and then to the final themes included frequent discussions with the principal investigator (CM). We used an analytic framework involving the identification of key concepts to discover core ideas, and to understand how participants viewed the topic [35]. MAXQDA 11 [36] software was used to assist with coding and analysis. MCU and BSM selected illustrative quotations. All quotations were translated into English by a native speaker. The consolidated criteria for reporting qualitative research (COREQ) (see Additional file 2) were used to report the findings of the study.

## Results

### Characteristics of participants and interviews

Forty-five patients were recruited by 13 general practices and 33 patients ultimately included in our study. The 12 remaining patients were not invited to participate as we noticed during our debriefings that no new topics arose in the course of interviews and focus groups. Two patients withdrew from the study (unavailable on the phone; cancelled the focus group). The final sample consisted of 16 women and 15 men. Sixteen telephone interviews (average duration 12.5 min) and two focus groups with the remaining 15 patients (average duration 1 h 12 min) were conducted. Table 1 displays the sample characteristics. Most patients reported they took six to seven medicines a day, always got them from the same pharmacy and had received a medication plan from their GP. None of the participants had previously received a structured medication review on a regular basis.

**Table 1:** Sample characteristics (*n* = 31)

	Patients (male/female) (n)	Mean age (years)	Range (years)
Telephone interviews	16 (10/6)	72 (SD 7,1)	(62–82)
Focus group 1	8 (3/5)	74 (SD 6,6)	(62–82)
Focus group 2	7 (2/5)	78 (SD 6,3)	(71–88)
All participants	31 (15/16)	74 (SD 7,0)	(62–88)

Each quotation is assigned a code: P stands for “patient”, and the number indicates the individual. An ellipsis surrounded by parentheses (…) denotes omitted text.

### Patient participation in an MR

#### Barriers

Some of our interviewees were sceptical about the need for MRs in general, or could understand why other patients might refuse participation. The three main reasons they gave concerned patient autonomy. The first point was satisfaction with the current medication regime and therefore reluctance to have anyone change it. The second point was an unwillingness to disclose all medicines during an MR. This unwillingness was mainly related to psychiatric treatment and over-the-counter (OTC) drugs. Patients wanted to decide for themselves when to speak about psychiatric diagnoses, and not to be forced to do so during an MR. Furthermore, OTC drugs are often not disclosed, sometimes because patients are unaware they can also lead to interactions, and sometimes because they fear having to justify what they take.

“I didn’t mention it either, that I take them. I didn’t think it was important because it’s herbal medicine (…).” (P 4, 66 years, telephone interview).

“Well, what they’re taking may not be that good, but when your sister has said, or your brother, that that’s ideal, you don’t want to be sort of dictated to … to be told what you have to take, when and why.” (P 9, 63 years, telephone interview).

The third point was that some patients disapproved of only having an MR at predetermined intervals. They agreed to have a review on a regular basis, e.g. once a year, but stressed that MRs should be available to them upon request, following hospitalisation, when new medicines are prescribed, or when medicines are changed. One patient, who was taking eight prescribed medicines per day, said he could not “wait half a year, or a year” (P24, 76 years, focus group 1) to talk to a doctor when he had a problem with his medication.

#### Facilitators

Most of our interviewed patients saw no reason to refuse an MR themselves, and regarded it as “definitely necessary that such a check is done” (P27, 76 years, focus group 2), not least because the majority of participants had already reflected upon the appropriateness of their medication and some had even undertaken measures to check it. These measures ranged from reading the patient information leaflet provided with their medicines, to preparing their own digital medication plan, or proactively asking their GP to check their medicines for potential interactions. These patients regarded a structured MR as a good opportunity to discuss their medications. Many patients could also imagine playing an active role by, for example, preparing a list of their medicines in advance, thus facilitating and shortening MRs.

During an MR, patients expected indications for their medicines to be checked, possible interactions with other drugs identified, information provided on the availability of new and better medicines, and possibilities to reduce the number of drugs they were taking to be investigated. Two main reasons for these expectations were revealed during interviews and focus groups. The first reason was medication-related somatic problems faced by the interviewees themselves, e.g. stomach bleeding or a restricted choice of headache pills when taking anticoagulants. The second reason was a more general distrust of the complex healthcare system and its various stakeholders. It was repeatedly seen as a problem that consulting several doctors (GPs and specialists) frequently led to uncoordinated prescription of multiple different medicines.

“If I could stop taking something - well I do take a lot - I should add that I really am all for stopping (...)” (P8, 77 years, telephone interview).

“Yes, especially concerning interactions. Do they agree with one another? Is it necessary that I take this kind of medicine? That’s really the main priority, and how they interact with one another. Yes, I always think; as many as necessary but as few as possible.” (P 12, 62 years, telephone interview).

### GP-led MRs

#### Barriers

The main arguments against GPs conducting MRs were a perceived lack of resources (financial and time constraints) on the one hand, and a lack of pharmacological competence on the other. Patients who were concerned that GPs would not have enough time for MRs based their doubts on the experience that the practice was always crowded. They feared a lack of time might prevent GPs from discussing patients’ medicines in the necessary detail. One patient questioned whether GPs would receive appropriate financial compensation. A few patients also doubted whether GPs had the necessary pharmaceutical expertise for an MR. However, such patients were never referring to their own GPs, and most of them were actually in favour of GP-led MRs.

“Well I reckon you're opening a can of worms - it’s definitely necessary that such a check is done - because I can’t imagine that all the things we swallow, that all those things agree with one another, but I don’t know if the GP has the ability or the time to do the research on it.” (P 27, 76 years, focus group 2).

#### Facilitators

The main facilitators to GP-led MRs were firstly his or her perceived pharmaceutical competence, and secondly the trusting relationship between patient and GP.

GPs were generally mentioned explicitly as the first point of contact in the event of medication-related problems. Most patients expected their GPs to have more medication-related experience and knowledge than pharmacists. They therefore concluded that GPs were best suited to conducting an MR.

“(…) when I get a new or a different medicine, I can read the description as often as I want, I still only understand 10% of it. Simple! I need someone that knows about it and who says, yes, that works or that doesn’t work. And if it’s not the GP, who sees me regularly and has known me for years, who is it going to be?” (P 21, 67 years, focus group 1).

“In that case I’d prefer the doctor. I don’t want to criticise the pharmacist. (…) I can just imagine the doctor knows more about side effects (…).” (P 13, 65 years, telephone interview).

Most of our interviewees reported having confidence in their GPs and regarded the trusting relationship with him or her as a good basis for a successful MR. They had often consulted the same GP for a long time. One participant also mentioned that his GP visited him at home as he was physically disabled. (Pharmacists in Germany do not currently perform home visits on a regular basis,) So he perceived he had “not many options” (P8, 77 years, telephone interview) other than a GP-led MR for practical reasons.

### Pharmacist-led MRs

#### Barriers

The main barrier to a pharmacist leading the MR was a lack of confidence in their pharmacological expertise. This mistrust was specifically described as resulting from personal experience when buying medicines in a pharmacy. When obtaining medicines, patients expected to be asked about illnesses and the other medications they were taking to ensure there was no danger of interactions. However, several patients reported that this had not always been the case. One 76-year-old patient, for instance, described reading the patient information leaflet after buying eye drops and discovering that his coronary heart disease was contra-indicated. Another 64-year-old patient said it upsets him for safety reasons that when buying aspirin, he was never asked whether he was taking anticoagulants. In consequence, he now mentions that he is taking anticoagulants when obtaining a medication from a pharmacy.

As a result, very few patients discussed problems with prescribed medicines with pharmacists but only consulted them when obtaining OTC drugs.

### Facilitators

Few patients saw any benefit in MRs being conducted by pharmacists. However, one patient mentioned that it was an advantage that some pharmacies already have digital records of the medicines taken by regular customers.

“That means that when I go to the pharmacy, all the medications I’m being prescribed are entered into a computer programme so that it’s possible to see, like I said, when I received what medication and in what dosage (…) Then they can see exactly what has been prescribed to date.” (P9, 63 years, telephone interview).

### Involvement of HCAs in MRs

#### Barriers

Several patients were unsure whether HCAs should be involved in MRs, generally because they were unsure whether HCAs received the necessary training.

“The problem is really to what extent the assistants can or are allowed to discuss such things. I don’t know what their training entails and whether they are competent enough for it (…) But if the doctor says Mrs. Whatever is capable enough then of course I would speak to her about it.” (P 2, 64 years, telephone interview).

#### Facilitators

Most patients found it acceptable that GPs should delegate parts of MRs to an HCA. They expected HCAs’ support to reduce the doctor’s workload and to facilitate the MR. As examples of such supportive tasks, participants said they could imagine HCAs calling up patients to make a list of their medications, or entering the medicines into a computer programme prior to the MR appointment.

## Discussion

### Main findings

Our study identified barriers and facilitators for four main topics regarding the implementation of MRs in primary care. These four topics comprised patient participation, GP-led MRs, pharmacist-led MRs, and involvement of HCAs in MRs.

Barriers to patient participation related to patient autonomy and included reluctance to change current drug regime, unwillingness to disclose psychiatric and OTC drugs, as well as disapproval of excessive rigidity in the intervals between MRs. However, as the majority of patients had already reflected upon the appropriateness of their medications, their awareness of medication-related problems was a facilitator. During an MR, patients expected indications for their medicines to be checked, possible interactions with other drugs identified, information provided on the availability of new and better medicines, and possibilities to reduce the number of drugs they were taking to be investigated. Barriers to GP-led MRs concerned GP’s lack of resources, whereas facilitators related to the trusting relationship between patient and GP. Respondents had differing views on the pharmaceutical competence of GPs. Although most had confidence in their doctor’s skill, some doubted he or she had sufficient pharmaceutical knowledge. Pharmacist-led MRs may be hindered by patients’ mistrust of pharmacists’ expertise, but could be facilitated by pharmacies’ digital records of a patient’s medicines. With regard to the involvement of HCAs in an MR, a potential barrier was that some patients doubted whether their training and competence were sufficient. However, most patients were happy with the idea that GPs would delegate some aspects of MRs to an HCA and thus facilitate their implementation.

### Findings in relation to the literature

Our findings are supported in the literature [15, 27, 28, 37] insofar as our patients’ attitudes towards MRs were mostly positive. Unlike as in some other studies, none of our patients viewed MRs negatively or as unnecessary due to the workload of existing medical appointments [26, 38]. Fears and suspicions that the purpose of MRs was to save money by stopping or changing medicines were mentioned by Petty et al. [27, 39]. Some of our patients also feared a change in a drug regimen they were satisfied with, but did not voice fears that the aim was to cut costs. Others found that additional drugs, such as herbal treatments, had only been documented by approximately 60% of GPs [40], and that some patients withheld information on these drugs from their doctor [41, 42]. Our interviewees also mentioned this problem. As inappropriate use can lead to potentially hazardous drug interactions, structured MRs should explicitly ask about additional drugs [43]. However, our findings suggest that patients need to be informed about the importance of disclosing additional drugs, as some of our interviewees perceived for instance herbal medicines as not important and never mentioned them to their GPs. Patients should not be forced to reveal all their medicines, but motivated to do so in a supportive manner.

Reports by patients that they had no opportunity to discuss medication-related questions and problems with a health professional can be found in the literature [15, 41]. These patients want to be properly informed about their medications, including possible side effects, and to receive detailed instructions on taking them [27, 44]. Such patients would undoubtedly appreciate the opportunity to discuss these issues in an MR [27, 45], and to have their questions answered [29], just like our interviewees. The wish to cease taking medications that are no longer necessary and to reduce the number of drugs being taken was a point mentioned in both telephone interviews and focus groups. Other authors have reported similar finding [46].

The majority of our patients had reflected upon the appropriateness of their medicines, and a few had even asked their GP to check their current medicines. However, in other studies, GPs’ lack of time and patients’ fears of wasting it were barriers to seeking help [47]. This may explain why many of our interviewees had not asked their GPs to review their medicines. Nonetheless, the inclusion of MRs in routine primary care would be welcomed by patients who are aware of potential problems and willing to do something about it, e.g. by seeking advice from journals, or on the internet [48, 49]. Our participants also undertook measures to check their medicines by, for instance, reading patient information leaflets. Our patients were able to imagine being actively involved in MRs. This could well be a facilitator, as patients would then be more likely to provide information on their actual drug use (including OTC drugs), adverse drug events, and practical and management problems [25].

Our interviewees welcomed the idea of GPs conducting MRs in preference to pharmacists. Other authors have also reported that patients welcomed MRs conducted by GPs [15, 27, 50]. However, considering the lack of research into patients’ views on MRs, it cannot be ruled out that many patients would be happy for pharmacists to perform them. Patients that had undergone an MR with a pharmacist spoke positively about it, and viewed the pharmacist as an expert on medicines [51]. Indeed, some of our interviewees could imagine that a pharmacist would conduct their MR.

### Strengths and limitations

One strength of our study is that some of our elderly patients expressed their opinions in telephone interviews and some in focus groups. Their average age was 74, and ranged from 62 to 88 years, enabling us to cover a wide spectrum of patients. Furthermore, our sample was balanced with respect to gender. As a result of our inclusion criteria, we selected patients who had most likely experienced medication-related problems, and could contribute to the discussion.

The study has several limitations. As we recruited patients in general practice, where patients generally have confidence in their GPs, our participants may have overemphasized the role of the GP in MRs. Furthermore, as we work at an institute of general practice, we may also have tended to overstate the role of GPs. To prevent this, we thoroughly discussed codes during the analysis. Studies inviting patients to community pharmacies might find slightly different attitudes. Although we conducted test interviews, our interview guide was not systematically pilot tested. Furthermore, patients could choose between telephone interview and focus group. The choice enabled us to lower the threshold for participation because housebound people could participate in a telephone interview. A similar study, by contrast, excluded housebound patients [27].

Patients participated voluntarily, so it is likely that they had a greater interest in MRs than the average patient. Furthermore, in the “project background” section of our interview guide, we state that taking many different medicines can cause problems. This may have encouraged patients to speak in more detail about their medication-related problems, as they felt confident that their problems were of interest to others. In general, participants’ statements have to be rated as assumptions – none had experienced a structured MR so far. Limitations usually associated with focus groups such as difficulties surrounding mutual self-disclosure on uncomfortable subjects may apply to our study as well [52]. However, in anticipation of this limitation we also conducted telephone interviews, which created a more personal atmosphere. Additionally, we used circular questions, inviting our interviewees to refer to other patients’ viewpoints, e.g. relatives or friends who had different experiences and problems [53]. Nevertheless, such viewpoints remain speculative.

## Conclusion

Our study suggests that patients regard MRs as beneficial and expect indications for their medicines to be checked, and possible interactions with other drugs to be identified. To foster the use of MRs in primary care, it is important to consider potential barriers and facilitators for the four different topics we identified. These topics comprised patient participation, GP-led MRs, pharmacist-led-MRs, and the involvement of HCAs.

These findings will be used to support the further implementation of our complex intervention on “PRIoritising MUltimedication in Multimorbidity in general practices (PRIMUM)” [22] to facilitate MRs in primary care. Furthermore, our findings may help in the design of MRs. According to our study, invitations should describe possible effects (e.g. reduction in number and complexity of medicines), and explicitly invite patients to consult their GPs. The structured MRs should also include questions on additional drugs, such as OTC medicines. Although our patients thought differently, we could envisage cooperation between GPs and pharmacists. Further research is needed here to identify and address possible obstacles from the point of view of all stakeholders. Our patients considered MRs to be necessary, but as resources in primary care are limited, further research should focus on precise inclusion criteria for the target group.

We confirm all patient/personal identifiers have been removed or disguised so that the patient/person(s) described are not identifiable and cannot be identified on the basis of the manuscript.

## Additional files

Additional file 1: Interview guide for telephone interviews and focus groups. (DOCX 22 kb)

Additional file 2: Consolidated criteria for reporting qualitative research (COREQ): a 32-item checklist for interviews and focus groups. (DOCX 19 kb)

## Abbreviations

ForN: Forschungsnetzwerk Allgemeinmedizin Frankfurt
GP: General Practitioner
HCA: Health care assistant
MR: Medication review
OTC: Over-the-counter
PRIMUM: PRIoritising MUltimedication in Multimorbidity in general practices
TK: Techniker Krankenkasse

## Footnotes

1 HCAs are medical assistants in the practice who receive less training than nurse practitioners and U.S. physician assistants and are comparable to certified medical assistants in the USA. They mainly perform administrative work and simple tasks such as weighing patients [54, 55]. HCAs have already been repeatedly and successfully involved in primary care-based, chronic care interventions in Germany. Under GP supervision, they have followed evidence-based protocols and algorithms with fixed interview questions, and provided telephone monitoring to check, for example, adherence to medication in patients with major depression, chronic heart failure, and osteoarthritis [56–58]
