# Supplementary material for: Patient-perceived barriers and facilitators to the implementation of a medication review in primary care: a qualitative thematic analysis
Source: BMC Fam Pract. 2018 Jan 5;19:3. doi: 10.1186/s12875-017-0707-0 (PMC5755323; doi:10.1186/s12875-017-0707-0)
Supplement: Supplementary file 1 — Interview guide for telephone interviews and focus groups. (DOCX 22 kb) [file 12875_2017_707_MOESM1_ESM.docx]

**Additional file 1: Interview guide for telephone interviews and focus groups**

## Greeting

Hello, my name is ________________. I am __________________ (description of what you do at the Institute).

Thank you for taking part in this telephone interview /discussion. It will take about 20 minutes / 1 – 1 ½ hours. Is it ok with you if we record the interview/discussion, so that we can better evaluate what you say?

We would like to ask you not to talk about our discussions outside these sessions, so that our view of things is not distorted in any way: it will create the trust that is necessary if everyone is to feel comfortable participating freely in the discussion. Furthermore, personal information about participants should not leave this room. Is that ok with you?

## Background of the project

Medicines are important in the treatment of illness. The older a person is, the more illnesses they tend to get. These often have to be treated with several medicines at the same time. Taking many different medicines can cause problems.

We have already conducted several preliminary studies aimed at making interactions between different medications safer. We have developed a medication review for family practices that examines whether medications are compatible or interfere with one another. Now we would like to see whether the medication review is suitable for everyday use, or whether there are barriers to its use that we did not previously recognize. We are currently in the planning phase and require your expertise.

*Instruction for interviewer: only mention content in parentheses if interviewee does not answer*

## Patients on multimedication

- What are your experiences of taking different medications?
- Who have you found you can turn to if you have questions relating to certain medications? What do other people you know do?
- Who do you normally turn to first?
- Do you always get your medications from the same pharmacist? What do other people you know do?

## Access

- If your health insurer were to select you for a possible medication review, they would not be permitted to write to your doctor but would have to contact you first. How would you prefer them to contact you? (write to you or to call you up, or perhaps both?)

## Extent of the intervention

Imagine you have an appointment for a medication review with your doctor, and that you are asked to bring all the medicines that you find at home with you, including old boxes etc., so they can all be looked at.

- Whom would you prefer to go through the medicines with you? (The HCA (health care assistant), the doctor, or both? And if both, in what order?)
- Could you imagine preparing a list of all your medicines at home? Could you imagine other patients would be able to do that?
- Could you imagine an HCA calling you up at home, so that you could go through all the medicines together and would not have to bring them all to the practice with you?
- What do you think is the maximum amount of time a medication review should take?
- In your opinion, how often should such reviews be carried out?
- Can you imagine there are any reasons why you or other patients would not be prepared to undergo a medication review?
- Can you imagine there are any reasons why you or other patients would not want to tell their doctors about certain medicines?
- How do you think you could benefit from such a medication review?
- What risks or problems do you think such a medication review might entail?

## Medication coach

- Some health insurance companies have pharmacists that provide telephone support to patients that are taking many different medicines. Have you heard anything about that?
- Could you imagine being called up and asked about your current medications by a pharmacist from your health insurance company?
- If you were to use such a service, how do you think it should be arranged? (combination of medication review, receiving a call before appointments at the practice etc.)
- Could you imagine that any other groups of patients exist that would benefit from such a call?

## Conclusion

- Is there anything you would like to add? Have we perhaps forgotten something that you think is important / that you would like to talk about?

Thank you for giving us so much of your time!
