# Supplementary material for: Patient-perceived barriers and facilitators to the implementation of a medication review in primary care: a qualitative thematic analysis
Source: BMC Fam Pract. 2018 Jan 5;19:3. doi: 10.1186/s12875-017-0707-0 (PMC5755323; doi:10.1186/s12875-017-0707-0)
Supplement: Supplementary file 2 — Consolidated criteria for reporting qualitative research (COREQ): a 32-item checklist for interviews and focus groups. (DOCX 19 kb) [file 12875_2017_707_MOESM2_ESM.docx]

**Additional file 2**

**Consolidated criteria for reporting qualitative research (COREQ): a 32-item checklist for interviews and focus groups**

Article: Patient-perceived barriers and facilitators to the implementation of a medication review in primary care: A qualitative thematic analysis
Authors: Mirella C Uhl, Christiane Muth, Ferdinand M Gerlach, Goentje-Gesine Schoch, Beate S Müller

# Domain 1: Research team and reflexivity

**Personal characteristics**

1. **Interviewer**

***Which author/s conducted the interview or focus group?***

Mirella Uhl and Beate Müller conducted the interviews and focus groups.

1. **Credentials**

***What were the authors’ credentials?***

Christiane Muth: MD, MPH

Ferdinand M Gerlach: Prof., MD, MPH

Goentje-Gesine Schoch: Pharm. D.

Beate S Müller: MD

1. **Occupation**

***What was their occupation at the time of the study?***

Mirella C Uhl: medical student and doctoral candidate

Christiane Muth: principal investigator

Ferdinand M Gerlach: Professor for general practice

Goentje-Gesine Schoch: pharmacist at Techniker Krankenkasse

Beate S Müller: academic GP

1. **Gender**

***Was the researcher male or female?***

First, second, fourth and fifth authors female, third author male.

1. **Experience and training**

***What experience or training did the researchers have?***

The last author had considerable experience in conducting interviews and focus groups, and briefed the first author carefully in advance of the study. Second, third and fourth authors all have had training and experience in conducting previous studies related to polypharmacy/multimorbidity and its management in primary care.

**Relationship with participants**

1. **Relationship established**

***Was a relationship established prior to study commencement?***

Patients had no previous association with the Institute.

1. **Participant knowledge of the interviewer**

***What did the participants know about the researcher?***

At the beginning of the interviews, the interviewers introduced themselves as staff from the Institute, but did not provide information on personal goals or academic background in order to minimize their influence on the conversation.

1. **Interviewer characteristics**

***What characteristics were reported about the interviewer?***

The potential for bias as we work at an institute of general practice and therefore may have tended to overstate the role of GPs was reported.

# Domain 2: study design

**Theoretical framework**

1. **Methodological orientation and Theory**

***What methodological orientation was stated to underpin the study?***

Thematic analysis.

**Participant selection**

1. **Sampling**

***How were participants selected?***

A convenience sample of patients was recruited.

1. **Method of approach**

***How were participants approached?***

GPs received consent forms and patient information sheets on the study, and gave them to the patients. After receiving written informed consent, the GPs informed the study center, the Frankfurt Institute of General Practice. Patients were then called up by the Institute to arrange appointments for either a telephone interview or a focus group session.

1. **Sample size**

***How many participants were in the study?***

The final sample consisted of 31 patients

1. **Non-participation**

***How many people refused to participate or dropped out? Reasons?***

45 patients were recruited and 33 ultimately included in our study. The 12 remaining patients were not invited to participate as we noticed during our debriefings that no new topics arose in the course of interviews and focus groups. Two patients withdrew from the study (unavailable on the phone; cancelled the focus group)

**Setting**

1. **Setting of data collection**

***Where was the data collected?***

Focus groups took place at the Institute of General Practice.

1. **Presence of non-participants**

***Was anyone else present besides the participants and researchers?***

No, there wasn’t.

1. **Description of sample**

***What are the important characteristics of the sample?***

Patients’ average age was 74 years (range 62-88 years). The sample consisted of 16 women and 15 men.

**Data collection**

1. **Interview guide**

***Were questions, prompts, guides provided by the authors? Was it pilot tested?***

Yes, the semi structured interview guide is described in detail in the Additional file 1. The interview guide was not systematically pilot tested.

1. **Repeat interviews**

***Were repeat interviews carried out?***

No.

1. **Audio/visual recording**

***Did the research use audio or visual recording to collect the data?***

Telephone interviews and focus groups were audio recorded.

1. **Field notes**

***Were field notes made during and/or after the interview or focus group?***

Yes.

1. **Duration**

***What was the duration of the interviews or focus group?***

Telephone interviews: average duration 12.5 min; focus groups: average duration 1 h 12 min.

1. **Data saturation**

***Was data saturation discussed?***

Yes.

1. **Transcripts returned**

***Were transcripts returned to participants for comment and/or correction?***

No.

# Domain 3: analysis and findings

**Data analysis**

1. **Number of data coders**

***How many data coders coded the data?***

Two.

1. **Description of the coding tree**

***Did authors provide a description of the coding tree?***

N/A

1. **Derivation of themes**

***Were themes identified in advance or derived from the data?***

Themes were identified from the data.

1. **Software**

***What software was used to manage the data?***

MAXQDA 11.

1. **Participant checking**

***Did participants provide feedback on the findings?***

No.

**Reporting**

1. **Quotations presented**

***Were participant quotations presented to illustrate the themes/findings?***

Yes.

***Was each quotation identified?***

They were not identified to preserve anonymity. However, each quotation is assigned a code: P stands for “patient”, and the number indicates the individual.

1. **Data and findings consistent**

***Was there consistency between the data presented and the findings?***

Yes.

1. **Clarity of major themes**

***Were major themes clearly presented in the findings?***

Yes.

1. **Clarity of minor themes**

***Is there a description of diverse cases or discussion of minor themes?***

Yes.
